# Peptide aptamer-modified single-walled carbon nanotube-based transistors for high-performance biosensors

**DOI:** 10.1038/s41598-017-18169-1

**Published:** 2017-12-20

**Authors:** Nguyen Thanh Tung, Phan Trong Tue, Truong Thi Ngoc Lien, Yasuhide Ohno, Kenzo Maehashi, Kazuhiko Matsumoto, Koichi Nishigaki, Manish Biyani, Yuzuru Takamura

**Affiliations:** 1 School of Materials Science, Japan Advanced Institute of Science and Technology, 1-1 Asahidai, Nomi city, Ishikawa 923-1292 Japan; 2 School of Engineering Physics, Hanoi University of Science and Technology, 1 Dai Co Viet Road, Hanoi, Vietnam; 3 Graduate School of Technology, Industrial and Social Sciences, Tokushima University, 2-4 Shinkuracho, Tokushima, 770-8501 Japan; 4 Institute of Engineering, Tokyo University of Agriculture and Technology, 2-24-16 Nakacho, Koganei, Tokyo 184-8588 Japan; 5 The Institute of Scientific and Industrial Research, Osaka University, 8-1 Mihogaoka, Ibaraki, Osaka 567-0047 Japan; 6 Department of Functional Materials Science, Saitama University, 255 Shimo-okubo Sakura-ku, Saitama city, Saitama 338-8570 Japan; 7 Center for Single Nanoscale Innovative Devices, Japan Advanced Institute of Science and Technology, 1-1 Asahidai, Nomi, Ishikawa 923-1292 Japan

## Abstract

Biosensors employing single-walled carbon nanotube field-effect transistors (SWCNT FETs) offer ultimate sensitivity. However, besides the sensitivity, a high selectivity is critically important to distinguish the true signal from interference signals in a non-controlled environment. This work presents the first demonstration of the successful integration of a novel peptide aptamer with a liquid-gated SWCNT FET to achieve highly sensitive and specific detection of Cathepsin E (CatE), a useful prognostic biomarker for cancer diagnosis. Novel peptide aptamers that specifically recognize CatE are engineered by systemic *in vitro* evolution. The SWCNTs were firstly grown using the thermal chemical vapor deposition (CVD) method and then were employed as a channel to fabricate a SWCNT FET device. Next, the SWCNTs were functionalized by noncovalent immobilization of the peptide aptamer using 1-pyrenebutanoic acid succinimidyl ester (PBASE) linker. The resulting FET sensors exhibited a high selectivity (no response to bovine serum albumin and cathepsin K) and label-free detection of CatE at unprecedentedly low concentrations in both phosphate-buffered saline (2.3 pM) and human serum (0.23 nM). Our results highlight the use of peptide aptamer-modified SWCNT FET sensors as a promising platform for near-patient testing and point-of-care testing applications.

## Introduction

The emergence of biosensor devices that convert a biological response into an electrical signal can address the rapidly increasing need in the point-of-care testing (POCT) market and achieve wide-scale application. However, the utilization of biosensor devices has yet to become mainstream, particularly for near-patient testing, mainly because of the difficulty in harnessing the combination of two key prerequisites: “sensitivity” and “selectivity”. To address the sensitivity issue, one-dimensional conductive nanomaterials such as carbon nanotube (CNT)-based field-effect transistors (FETs) have emerged as an effective transducer for label-free nanoelectronic platforms because of their exquisite electrical sensitivity toward minute variations in their surrounding environment^1–4^. However, the use of CNT-FETs in practical applications in clinical diagnostics remains a challenging issue because the obvious essential requirement of POCT is to recognize targets specifically in the presence of thousands-fold excesses of interfering species within a complex biological sample. For example, human serum contains hundreds of thousands of different protein molecules, of which 97% are composed of the 20 most abundant proteins^5^. Therefore, in this work, our intention is to focus on the selectivity issue while maintaining the greater sensitivity of semiconductor electronics devices. Because the large specific surface area (SSA) in CNTs, which enables immobilization of a large number of functional units at the carbon nanotube surface, increases with a decreasing number of walls, we preferred to use single-walled carbon nanotubes (SWCNTs) to improve the signal-to-noise ratio.

The bioelectronics interface interaction of CNTs with biological molecules has quite recently become the topic of focus after the discovery of a monoclonal antibody^6^ and peptide^7^ capable of selectively binding to the surface of CNTs. Interestingly, a dodecapeptide capable of self-assembly into peptide nanowires on graphene has recently been reported^8^. Therefore, peptide-based probes enable new approaches to develop CNT-based biosensors. To take a further step, our philosophy is to develop a new generation of biosensing technology by introducing evolutionary molecularly engineered peptide aptameric reagents^9^ as a smart biorecognition element onto SWCNT FETs to combine the high sensitivity of SWCNTs with the outstanding binding properties of the peptide aptamer to achieve excellent selectivity. In our previous research, we fabricated DNA aptamer-based CNT-FETs and evaluated the detection of IgE with higher sensitive performance^10,11^. However, peptide-based aptamers, which are comparatively smaller in molecular weight, exhibit a smaller binding footprint, allowing for a more thorough and precise interrogation of the target than that afforded by nucleic acid-based aptamers^12^. Most importantly, a peptide aptamer can be engineered on-demand through an *in vitro* selection procedure and can thus be targeted to the detection of biological molecules that are fundamentally unable to be detected via conventional approaches. Besides, antibody-based recognition elements with heights greater than 10 nm remain outside the electrical double layer (Debye length) and are thus unfit for use in a SWCNT FETs biosensor^13,14^. Interestingly, the smaller size of a peptide aptamer (less than a few nanometers) enables the detection of proteins in solutions of high ionic strength (i.e., approaching the ionic strengths found in physiological environments) and beyond the electrical double layer using SWCNT FETs.

**Figure 1:**
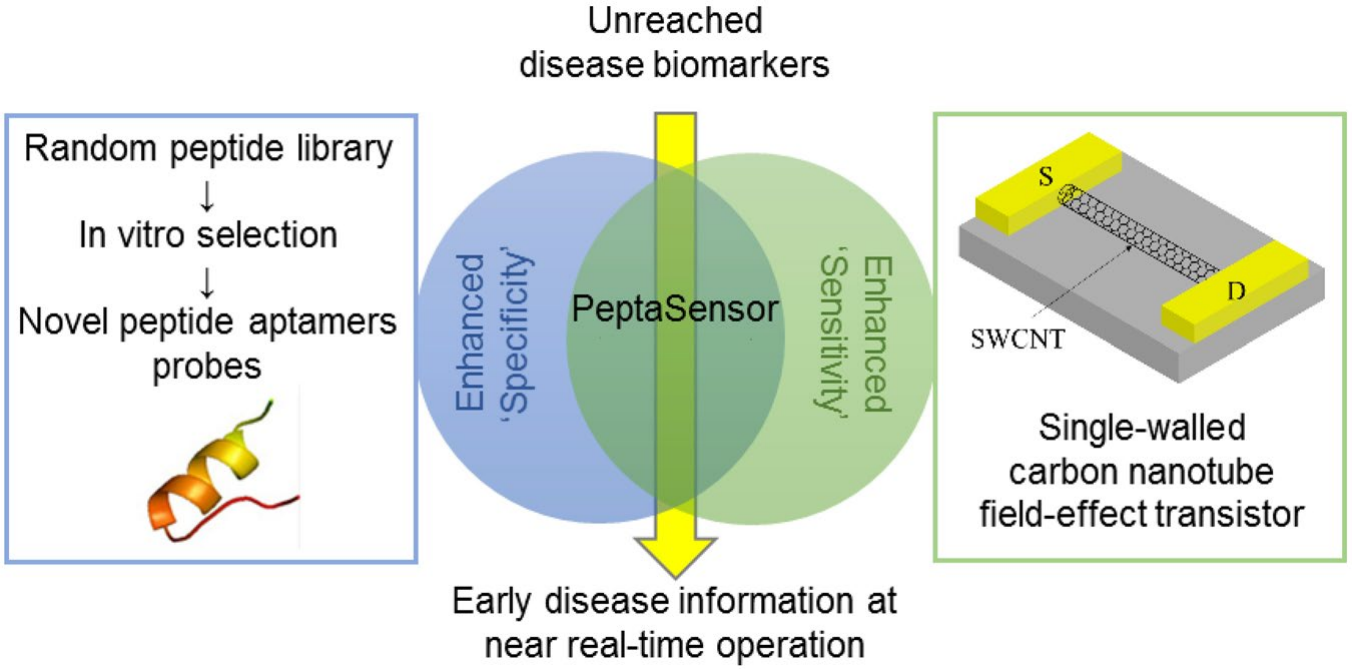
Schematic of the concept. The integration of a novel peptide aptamer with a SWCNT FET to achieve highly selective and sensitive biosensing of unreached biomarkers.

Recently, the application of repeats of amino acids integrated with CNT FET sensor has been developed for the detection of heavy-metal ions such as Ni^2+^, Cu^2+^ ^15^, and environmentally toxic chemicals such as decabrominated diphenyl ether (DBDE)^16^ and 2,4,6-Trinitrotoluene (TNT)^17^. In this work, we fabricated SWCNT FETs and investigated the integration and application of an engineered peptide aptamer for the highly selective and sensitive detection of unreached biomarkers for POCT application (Fig. 1). To do so, we modified the channels of SWCNT FETs by novel peptide aptamers that were engineered with a selective affinity for Cathepsin E (CatE) via systemic *in vitro* evolution^18^. We identified an alpha-strand peptide scaffold and conjugated it to a novel peptide aptamer with high binding affinity (0.954 nM) for CatE. This conjugated peptide was then grafted onto an SWCNT via noncovalent bonding. As a result, this FET device provides a strong yet selective capture of target CatE molecules; consequently, extremely sensitive detection of CatE can be achieved using this FET sensor system. Our device exhibited good selectivity (no response for bovine serum albumin (BSA) and cathepsin K (CatK)) and a low detection limit. The sensitivity of this device could improve the limit of detection of CatE in human serum by at least three orders of magnitude compared with a conventional ELISA-based system using a similar peptide aptamer^19^.

This work details the successful fabrication and demonstration of a liquid-gated SWCNT FET sensor modified with CatE-binding peptide aptamers as an attractive platform for POCT application for the detection of unreached disease biomarkers such as serum CatE, which has recently been highlighted as a novel prognostic biomarker for cancer because the reduction of serum level of CatE has been associated with poor prognosis in breast cancer patients^20^. To the best of our knowledge, this work represents the first report of an FET-type sensor utilizing peptide aptamer-modified SWCNTs; this novel sensor can lead to the development of a portable and affordable sensing system for high-performance near-patient testing.

## Results and Discussion

### Characteristics of the fabricated SWCNT FETs

**Figure 2:**
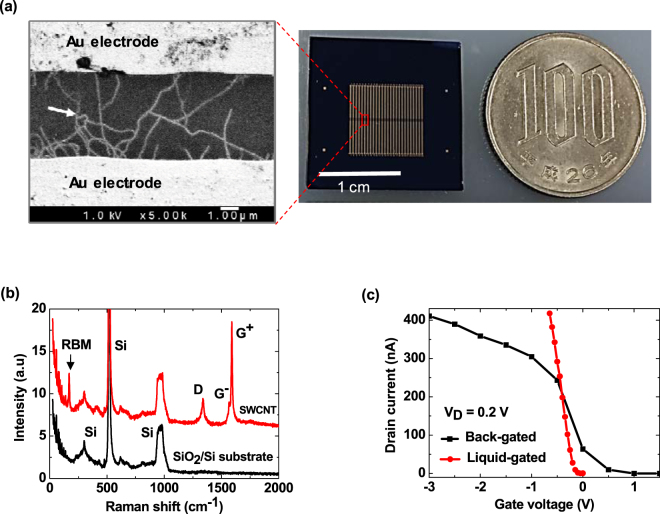
Device fabrication and characterization. (**a**) SEM image of a fabricated SWCNT FET (white arrow indicates the SWCNT) and real image of a fabricated chip, which contains an array of 52 SWCNT FETs; (**b**) Raman spectra of an as-grown SWCNT and the substrate (black arrow indicates the characteristic RBM of the SWCNT); and (**c**) transfer characteristics of the fabricated SWCNT FET.

Figure 2a shows a photo of a fabricated chip, which contains an array of 52 SWCNT FETs. In the FET, SWCNTs function as a semiconducting channel. A scanning electron microscopy (SEM) image of a fabricated SWCNT FET shows that several CNTs bridge two Au electrodes, indicating successful fabrication of the device (Fig. 2a). The growth of these CNTs was facilitated by a Co catalyst. To confirm the structure of the synthesized CNTs, Raman spectra of the as-grown CNTs and a SiO_2_/Si substrate were recorded, as shown in Fig. 2b. A single radial breathing mode (RBM) was observed in the Raman spectrum of the CNTs, indicating that the SWCNTs were successfully synthesized using the chemical vapor deposition (CVD) method. This characteristic RBM observed at 170 cm^−1^ corresponds to an as-grown SWCNT that is 1.4 nm in diameter^21^.

Transfer characteristics of the fabricated SWCNT FET were measured using back-gated and top liquid-gated schemes (Fig. 2c). The back-gate bias in air was swept from −3.0 to 1.5 V. The liquid-gated bias was scanned from 0 to −0.6 V. Such a small liquid-gated voltage was used to prevent undesirable electrochemical reactions between the source/drain metal electrodes and the liquid^10^. In both measurements, the drain currents decreased with increasing gate voltage, indicating that hole conduction was dominant in the fabricated SWCNT FET. A similar tendency of the drain current versus gate voltage was also observed in the output characteristics (see Supplementary Fig. S1). The top liquid-gated device exhibited a smaller subthreshold swing factor (S) (31 mV/decade) compared with that of the back-gated device (337 mV/decade). This small S-factor of the liquid-gated SWCNT FET was due to a large capacitance induced by the formation of an ultrathin electrical double layer in the vicinity of the SWCNT channel^22^. Generally, in the case of FET-based biosensors, a smaller S-factor indicates greater sensitivity. Furthermore, the performance of the top liquid-gated device with respect to repeated measurements was much more stable than that of the back-gated device (see Supplementary Fig. S2). This stability stems from the SWCNT channel being covered by the liquid, thereby preventing the effects of the surrounding environment. In addition, the use of an Ag/AgCl reference electrode can minimize voltage fluctuations. These stability properties are critically important for realizing reliable biosensing devices.

### Characterization of the peptide aptamer probe

**Figure 3:**
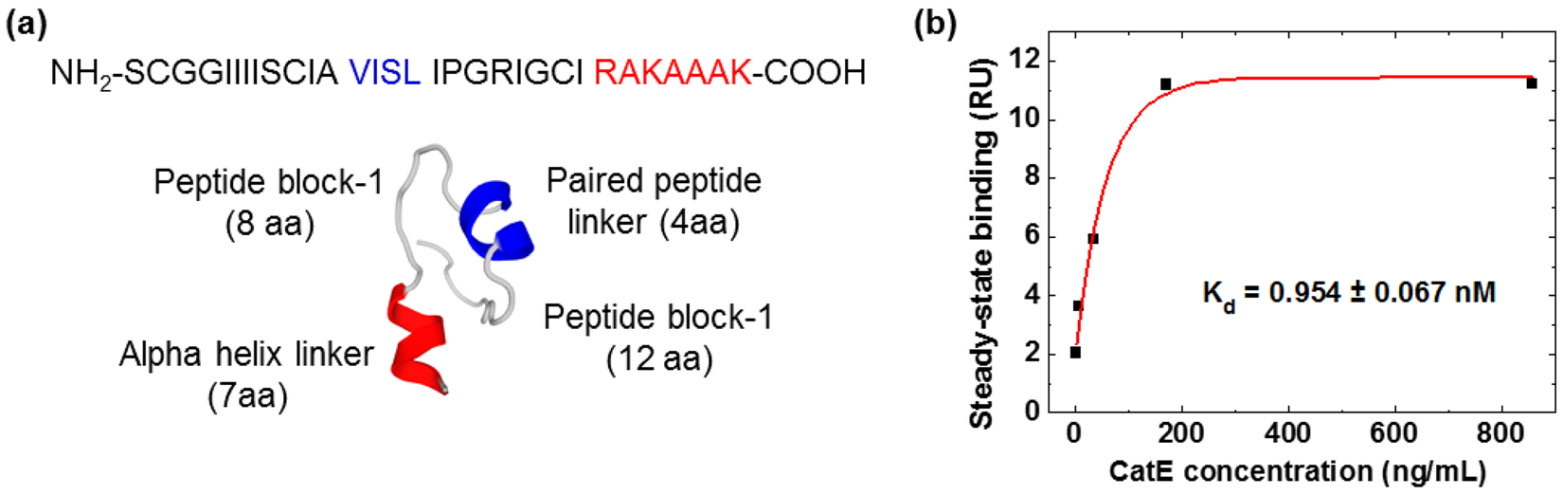
Peptide aptamer characterization. (**a**) The sequence and predicted structure of the selected peptide aptamer; and (**b**) SPR analysis for the affinity binding of the peptide aptamer to Cathepsin E using single-cycle mode with an aptamer level of 1000 RU and sequential injections of five ascending concentrations of analyte Cathepsin E (858.37, 171.67, 34.33, 6.87, 1.37 ng/mL). The data for the steady-state affinity binding plot were calculated from the end of the association phases against the analyte concentration.

A *de novo* simulated model of the peptide aptamer with selective affinity for CatE is shown in Fig. 3a. CatE is composed of an *in vitro*-selected peptide aptamer (24 amino acids comprising two peptide blocks of 12- and 8-amino acids paired by a 4-amino acid linker) and a short alpha-strand peptide scaffold (7-amino acids). A Biacore X100 instrument was used to characterize the binding features of the peptide aptamer to CatE. A free N-terminal end of the peptide aptamer is necessary for its correct folding as a basis for the formation of the binding complex with the target. Therefore, the C-terminal end of the peptide aptamer with biotin-streptavidin chemistry was immobilized onto a CM5 sensor chip, and then CatE was injected as the analyte across this surface for interaction with the peptide aptamer. Figure 3b shows the result of the Biacore measurement of the peptide aptamer. The dissociation constant (K_d_) for the CatE was calculated as 0.954 ± 0.067 nM.

### Quantitative detection of CatE in PBS buffer using the peptide aptamer-modified SWCNT FET biosensor

After fabricating the SWCNT FET, we immobilized the CatE-binding peptide aptamer onto the SWCNT channel via a 1-pyrenebutanoic acid succinimidyl ester (PBASE) linker to develop a peptide aptamer-modified SWCNT FET sensor for CatE detection. The immobilization of the PBASE linker and peptide aptamer were systematically investigated (see Supplementary Fig. S3). The relative decrease in the “on” current (∆I/∆I_max_), where ∆I_max_ represents the decrease in the “on” current at a saturation level due to the interaction between PBASE and SWCNT FETs, increased with increasing PBASE concentration from 3 to 50 mM and appeared to be saturated at 50 mM (Fig. S3a). The charge transfer induced by π-π interaction between the PBASE and SWCNT might affect the hole carrier inside the SWCNT channel, leading to the observed decrease in ∆I/∆I_max_^23^. This observation indicates the success of the PBASE linker immobilization. Therefore, 50 mM PBASE was selected for the subsequent experiments. Supplementary Fig. S3b shows that the relative decrease in the “on” current (∆I/∆I_max_), where ∆I_max_ represents the decrease in the “on” current at a saturation level due to the interaction between peptide aptamers and SWCNT FETs, increased with increasing peptide aptamer concentration in the range from 1 to 120 µM and then became saturated near 120 µM. The immobilization time of 60 min was selected as the optimal condition because aggregation of the peptide aptamer occurred at longer incubation times. The ∆I/∆I_max_ indicated the successful immobilization of the peptide aptamer probe molecules onto the SWCNT channel. The peptide aptamer, which has an isoelectric point of 9.77, exhibits positive charge in PBS pH 4. This positive charge of the peptide aptamer might affect the hole carrier inside the SWCNT channel, leading to the ∆I/∆I_max_. A large coverage of peptide aptamer over the SWCNT channel is critically important because it maximizes the target capturing efficiency. Therefore, 120 µM was chosen as the optimal concentration of the peptide aptamer. The fabricated sensors could be stored in the dried form at cool temperature of less than or equal to 4 °C.

**Figure 4:**
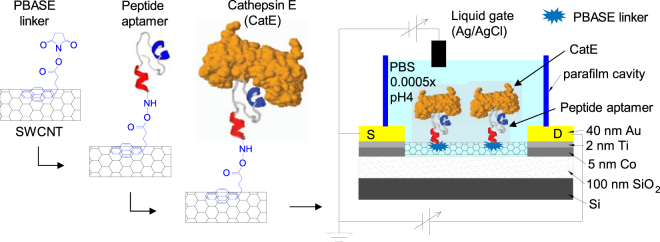
Schematic of the experimental setup. The immobilization of the peptide aptamer onto the surface of a SWCNT and the operating setup of the liquid-gated SWCNT FET device for CatE detection.

**Figure 5:**
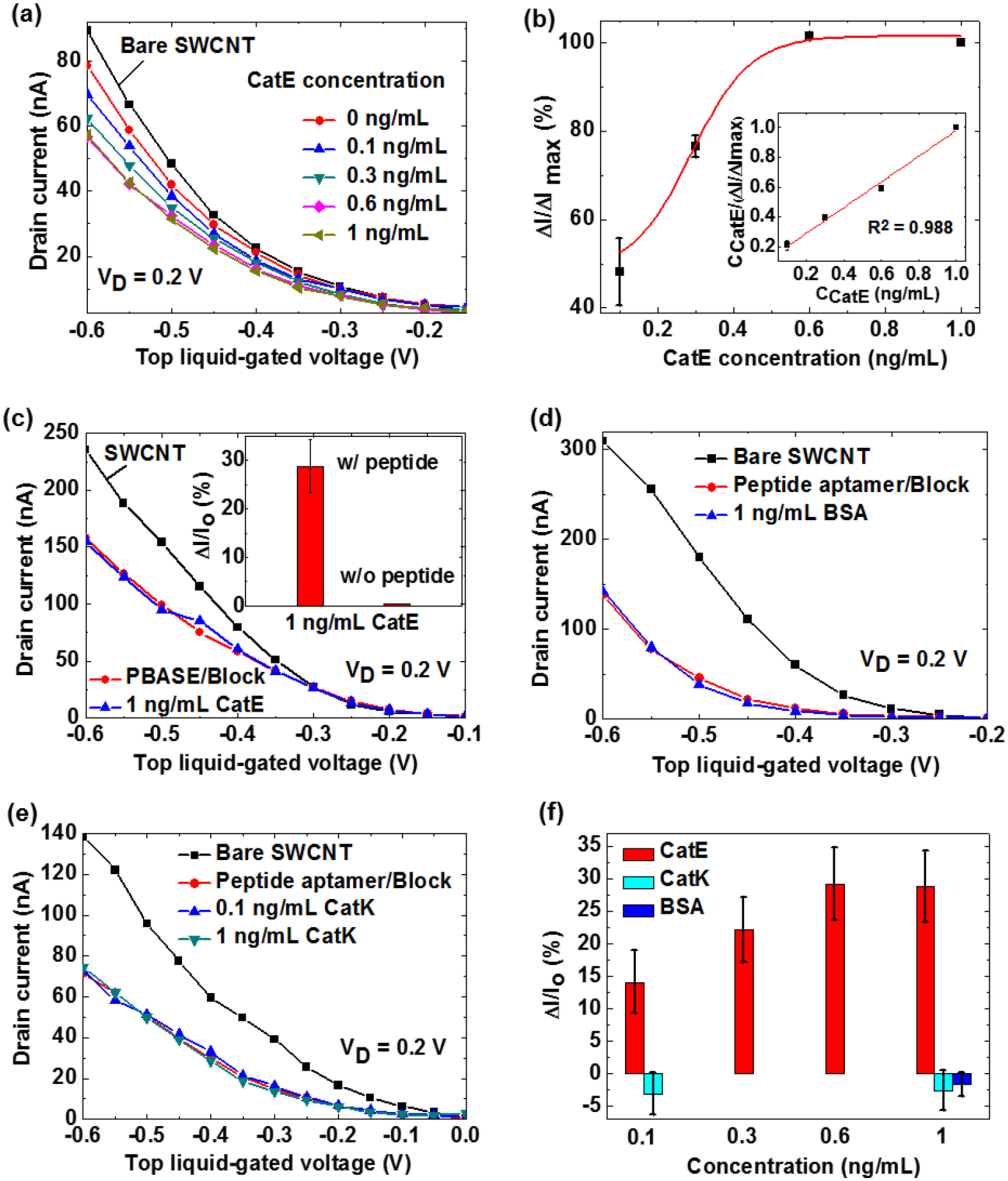
Quantitative detection of CatE in phosphate-buffered saline. (**a**) Transfer characteristics of the peptide aptamer-modified SWCNT FET for various CatE concentrations. (**b**) The relative decrease in the “on” current (∆I/∆I_max_) as a function of the CatE concentration. The inset shows the CatE concentration/(∆I/∆I_max_) as a function of CatE concentration. (**c**) Transfer characteristics of the PBASE-modified SWCNT FET without the peptide aptamer for 1 ng/mL CatE. The inset shows the comparison of the SWCNT FETs with and without peptide aptamers responding to 1 ng/mL CatE. Transfer characteristics of the peptide aptamer-modified SWCNT FET for 1 ng/mL BSA (**d**) and 0.1 and 1 ng/mL CatK (**e**). (**f**) The relative change in “on” current (∆I/I_o_) versus concentration was plotted for CatE, BSA and CatK.

A schematic of the setup for CatE detection using the peptide aptamer-modified SWCNT FET is shown in Fig. 4. The peptide aptamer was immobilized onto the SWCNT channel via the PBASE linker prior to the capture of CatE. Next, the sensor was exposed to 0.0005× PBS pH 4 for electrical measurements. Figure 5a shows the transfer characteristics of the peptide aptamer-functionalized SWCNT FET biosensor for various CatE concentrations ranging from 0.1 to 1 ng/mL. The transfer curve of the SWCNT FET initially shifted toward the negative bias direction after the immobilization of the peptide aptamer. With increasing CatE concentrations from 0.1 to 0.6 ng/mL, the curve further shifted toward the same direction. This phenomenon is attributed to the positively charged CatE molecules at PBS pH 4 (the isoelectric point of CatE is 4.69) inducing the gradual reduction of hole carriers in the SWCNT channel via a field effect^24^. At concentrations greater than this concentration, the shift of the transfer curve is no longer observed because all peptide aptamer probe molecules are occupied by CatE molecules. The relative decrease in the “on” current (∆I/∆I_max_), where ∆I_max_ represents the decrease in “on” current at a saturation level due to the interaction between CatE and peptide aptamer-modified SWCNT FETs, at a top liquid-gated voltage of −0.6 V was plotted as a function of CatE concentration, as shown in Fig. 5b. The results reveal that ∆I/∆I_max_ increased as a function of CatE concentration from 0.1 to 0.6 ng/mL and then saturated with a further increase of the CatE concentration. The minimal detectable concentration of 0.1 ng/mL is three orders of magnitude lower than that for a previously reported CatE biosensor^25^. The inset of Fig. 5b shows the dependence of CatE concentration C_CatE_/(∆I/∆I_max_) as a function of CatE concentrations. The good linear fitting of the experimental results indicates that the capture of CatE on the peptide aptamer-functionalized SWCNT is in accordance with the Langmuir adsorption isotherm given by equations (1) or (2):

1 $$({\rm{\Delta }}{\rm{I}}/{{\rm{\Delta }}{\rm{I}}}_{{\rm{\max }}})/{({\rm{\Delta }}{\rm{I}}/{{\rm{\Delta }}{\rm{I}}}_{{\rm{\max }}})}_{{\rm{\max }}}={{\rm{C}}}_{{\rm{CatE}}}/({{\rm{K}}}_{{\rm{d}}}+{{\rm{C}}}_{{\rm{CatE}}})$$

2 $${{\rm{C}}}_{{\rm{CatE}}}/({\rm{\Delta }}{\rm{I}}/{{\rm{\Delta }}{\rm{I}}}_{{\rm{\max }}})={{\rm{C}}}_{{\rm{CatE}}}/{({\rm{\Delta }}{\rm{I}}/{{\rm{\Delta }}{\rm{I}}}_{{\rm{\max }}})}_{{\rm{\max }}}+{{\rm{K}}}_{{\rm{d}}}/{({\rm{\Delta }}{\rm{I}}/{{\rm{\Delta }}{\rm{I}}}_{{\rm{\max }}})}_{{\rm{\max }}}$$

where K_d_ is the dissociation constant of the reaction between CatE molecules and the CatE-specified peptide aptamer probe molecules and (∆I/∆I_max_)_max_ is the saturated amount of the relative decrease in the “on” current. From the fitting, K_d_ was estimated to be 0.12 ng/mL or 2.769 pM, which is approximately 2 order smaller than that estimated from the surface plasmon resonance (SPR)-based titration (40.9 ng/mL or 0.954 nM). This difference is attributed to the difference in the range of CatE concentrations. In case of using SPR, the range of CatE was from 1.37 to 858.37 ng/mL. In case of using FET, the range of CatE was from 0.1 to 1 ng/mL. Additionally, the hydrophobic interaction between the SWCNT and the CatE may affect to the decrease in the dissociation rate constant, resulting in the smaller dissociation constant K_d_.

Figure 5c shows the result for the PBASE-modified SWCNT FET without the peptide aptamer probe molecule for CatE detection. There was almost no change observed in the drain current. The inset in Fig. 5c shows the comparison of the SWCNT FETs with and without peptide aptamer probe molecules responding to 1 ng/mL CatE. The normalization of “on” current (∆I/I_o_), where I_o_ represents the “on” current in presence or absence of target, was used for this comparison. The apparent change in (∆I/I_o_) was observed in case of SWCNT FETs with peptide aptamer while negligible change was observed in case of SWCNT FETs without peptide aptamer. This result indicates that the non-specific binding of CatE was successfully suppressed. Figure 5d and e show the results for the response of the peptide aptamer-modified SWCNT FET biosensor for BSA and CatK detections, respectively. The changes in the drain currents were hardly observed in both cases, indicating that the device was able to distinguish among the CatE target and interfering BSA, CatK molecules. By using the normalization of “on” current (∆I/I_o_), where I_o_ represents the “on” current in presence or absence of target, the responses of the peptide aptamer-modified SWCNT FETs to concentration of CatE, BSA and CatK were plotted as shown in Fig. 5f. The obvious changes in ∆I/I_o_ were observed after the introduction of CatE concentration while the small changes in ∆I/I_o_ in the opposite direction were observed after the introduction of BSA as well as CatK. These results suggest that high selectivity originated from the specific binding of the CatE target and peptide aptamer probe molecules, as anticipated from the Biacore measurements.

Supplementary Fig. S4 shows the atomic force microscopy (AFM) images of a bare SWCNT, a peptide aptamer-immobilized SWCNT, and a CatE-captured SWCNT. No change in the height of the SWCNT before (Fig. S4a) and after peptide aptamer immobilization (Fig. S4b) was observed. The peptide aptamer molecules with the short length of 31 amino acids might lie down on the sensor surface, as measured in a dried form, resulting in no difference in the observed height. By contrast, the height increased from 1.5 nm to 2.5 nm after CatE addition (Fig. S4c), which further confirmed that CatE molecules were captured by the peptide aptamer probe molecules.

### Quantitative detection of CatE in human serum

**Figure 6:**
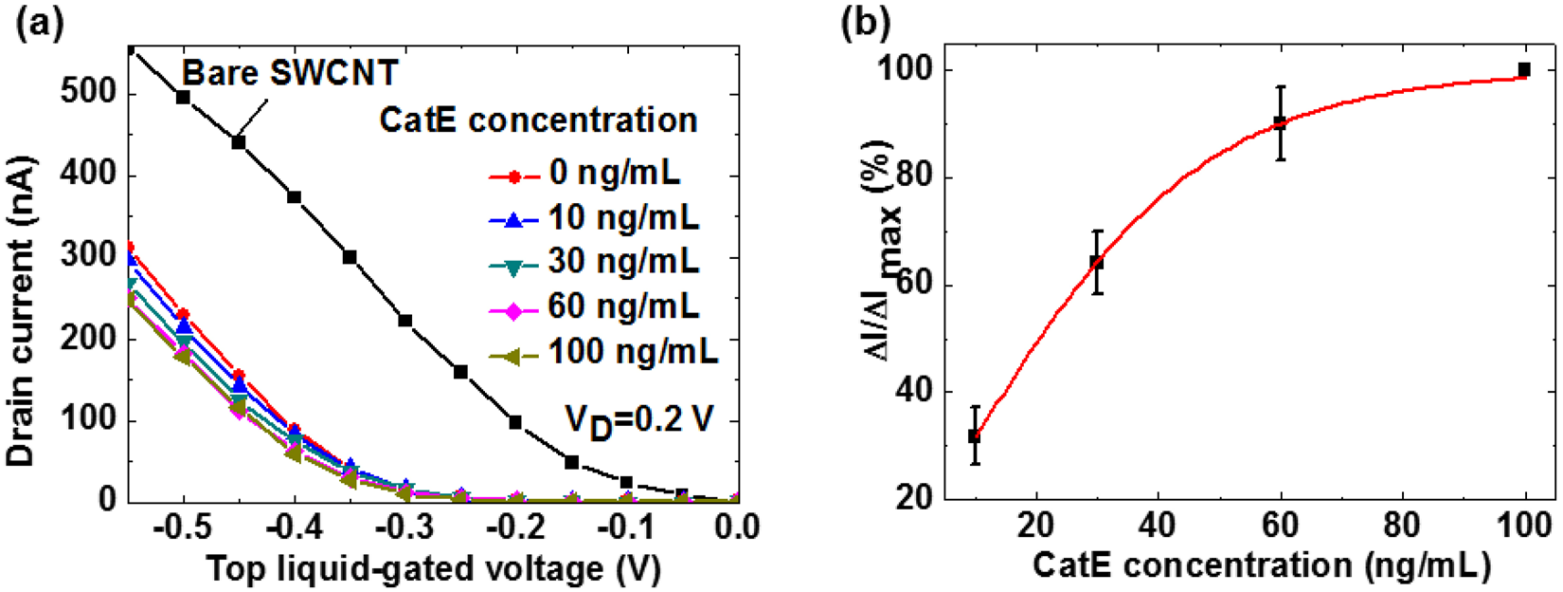
Quantitative detection of CatE in human serum. (**a**) Transfer characteristics of the peptide aptamer-modified SWCNT FET for various CatE concentrations in 10-fold-diluted human serum. (**b**) The relative decrease in “on” current (∆I/∆I_max_) as a function of CatE concentration.

From a point-of-care perspective, the direct and facile measurement of a cancer biomarker in blood serum is necessary. However, in the absence of a blocking treatment, a certain amount of response can be expected because of the non-specific adsorption or noise generated by bulk proteins present in human serum. To reduce such non-specific adsorption, we introduced the well-known blocking reagent, ethanolamine^26^. We further explored the potential application of our biosensor to detect CatE in a human serum sample by measuring the transfer characteristics of the peptide aptamer-modified SWCNT FET as a function of the CatE concentrations (from 10 to 100 ng/mL) in 10-fold diluted human serum (Fig. 6). Similar to the observations for the device in the PBS buffer, the transfer curve of the SWCNT FET initially shifted toward the negative bias direction after immobilization of the peptide aptamer and the addition of CatE target with concentrations from 10 ng/mL to 60 ng/mL (Fig. 6a). However, no response was observed above the CatE concentration of 60 ng/mL. This result indicates that the limit of detection in the presence of 10-fold diluted serum (i.e., 10 ng/mL) was impaired by two orders of magnitude compared with the limit in the presence of PBS (i.e., 0.1 ng/mL). A calibration curve was obtained as a function of serum CatE concentration using the normalization of the relative decrease in “on” current (∆I/∆I_max_), where ∆I_max_ represents the decrease in “on” current at a saturation level due to the interaction between serum CatE and peptide aptamer-modified SWCNT FETs (Fig. 6b).

The other (contaminating) proteins in human serum may affect responses in two ways. First, the non-specific adsorption of charged proteins onto the PBASE-modified SWCNT surface may lead to a shift in the transfer curve. In addition, the non-specific proteins, which are larger, may be adsorbed or hindered from moving onto the surface of the probe molecules and thus hide the recognition/binding site of the peptide aptamer, leading to a decrease in the response caused by the target protein. To address these challenges, a microfluidic approach can be used to reduce the possible non-specific interactions. Our sensor provided an improvement of sensitivity of serum CatE by at least three orders of magnitude compared to a conventional ELISA system using a similar peptide aptamer^19^. Given this observation and with implementation of further improvements in the sampling process, our peptide-modified SWCNT FET biosensor could offer an attractive approach to detect CatE in a real clinical sample with higher selectivity and the desired sensitivity.

## Conclusion

We presented the first demonstration of a peptide aptamer-modified SWCNT FET-based biosensor for highly selective and sensitive detection of CatE in serum. This device was realized by the combination of a novel peptide aptamer as a probe molecule and a high-performance SWCNT FET as a transducer. The minimal detectable CatE concentration of 10 ng/mL in human serum was achieved; this value is three orders of magnitude lower than that of the conventional ELISA method using the same peptide aptamer. The high specificity originates from selective binding of the peptide aptamer to the target CatE. Our results suggest that the integration of a highly specific peptide aptamer and a SWCNT FET transducer can be a platform for near-patient detection of biomarkers and thus could be widely used for human health monitoring and early disease diagnosis, such as breast cancer diagnosis.

## Methods

### Materials and apparatus

Ethanol (super dehydrated, 95%) and dimethylformamide (DMF) (98%) were purchased from Wako Pure Chemical Industries, Ltd., Japan. The 1-pyrenebutanoic acid succinimidyl ester (PBASE) linker was purchased from Thermo Fisher Scientific Inc., Japan. The peptide aptamer with a sequence of SCGGI IIISC IAVIS LIPGR IGCIR AKAAAK that specifically recognizes CatE was custom-made by Scrum, Inc., Japan. CatE was purchased from BioVision, Inc. (CA, USA). CatE samples were prepared in 1× PBS pH 10 and 10-fold-diluted human serum, which was diluted with 1× PBS pH 7.4 and then stored at −20 °C until use. Cathepsin K Active human and BSA, which were purchased from Sigma-Aldrich Co. LLC., Japan and Wako Pure Chemical Industries (Tokyo, Japan), respectively, were prepared in PBS and then stored at −20 °C. The 0.0005× PBS pH 4 was prepared by diluting 10× PBS with milli-Q water and then adding HCl to adjust the pH value to 4. A 10× PBS stock solution was prepared by adding 80 g NaCl, 2 g KCl, 14.4 g Na_2_HPO_4_ and 2.4 g KH_2_PO_4_ to 1 L milli-Q water. SEM and Raman spectroscopy methods were used to characterize the SWCNTs. A Biacore X100 instrument was used to characterize the binding features of the peptide aptamer to CatE. The AFM method was used to visualize the capture of CatE by the peptide aptamer, which was immobilized onto the SWCNTs. The Agilent 4156 C Precision Semiconductor Parameter Analyzer was utilized for measuring the electrical performance of the pristine SWCNT FET and the electrical signals of peptide aptamer-modified SWCNT FET induced from binding events.

### Fabrication of SWCNT FET

We fabricated an array of SWCNT FETs onto a heavily doped p^+^-Si substrate capped with a 100-nm-thick thermally grown SiO_2_ layer; the p^+^-Si substrate was used as a back-gate electrode^10,11,27^. First, the Si_2_O/Si substrate was cleaned with piranha solution for 10 min, rinsed with milli-Q water, and then dried with a flow of nitrogen gas. The Co catalyst film (5 nm) was deposited using electron-beam evaporation and then patterned via the photolithography technique. The SWCNTs were then grown on the Co catalyst patterns using the thermal CVD method at an ethanol pressure of 300 Pa (as carbon source) and temperature of 850 °C for 30 min (see Supplementary Fig. S5). Afterwards, fabrication of Ti (2 nm)/Au (40 nm) films as the source and drain electrodes was performed using a conventional photolithography and lift-off process (see Supplementary Fig. S6). The channel length was designed as 3 µm.

### Peptide aptamer characterization

A novel peptide aptamer that specifically recognizes CatE was engineered via systemic *in vitro* evolution. We characterized the binding affinity of the peptide aptamer and CatE using a Biacore X100 instrument. The SPR analysis for the affinity binding of the peptide aptamer to CatE used a single-cycle mode with an aptamer level of 1000 RU and sequential injections of five ascending concentrations of analyte CatE (858.37, 171.67, 34.33, 6.87, 1.37 ng/mL). A steady-state affinity binding plot was calculated from the end of the association phases against the analyte concentration.

### Surface modification of the SWCNT FET and CatE detection scheme

The CatE-specified peptide aptamer probe molecule was immobilized onto the SWCNT channel of the SWCNT FET via the PBASE linker. First, the PBASE linker was immobilized onto the SWCNT channel by dropping 50 mM PBASE solution onto the device and then incubating for 1 h at room temperature. Afterwards, 120 µM peptide aptamer dissolved in DMF was dropped onto the device, which was then incubated for 1 h at room temperature, rinsed thoroughly with DMF and then dried with nitrogen gas. The PBASE can bind by noncovalent π-stacking with the sidewall of SWCNTs via its aromatic ring. The peptide aptamer probe molecule covalently binds to the linkers via its succinimidyl ester functional group. The unreacted PBASE linker was blocked with 100 mM ethanolamine for 30 min. The devices were subsequently subjected to various CatE concentrations (from 0.1 to 1 ng/mL) in 1× PBS pH 10, incubated for 10 min at 25 °C to capture the CatE target, rinsed thoroughly with 0.0005× PBS pH 4 and then dried with nitrogen gas. Finally, a Parafilm cavity was attached to the device to contain the PBS buffer solution during electrical measurements.

We measured the transfer characteristics of the fabricated sensor using a top liquid-gated scheme in 0.0005× PBS pH 4. The source electrode was grounded. A 0.2 V bias voltage was applied between the source and drain electrodes to monitor the electrical conductance of the SWCNT channel while a top liquid-gated potential with respect to the grounded source electrode was scanned from 0 to −0.6 V. A reference electrode (Ag/AgCl, Bioanalytical Systems, West LaFayette, IN) was used to apply potential to the top liquid gate to avoid sensing an artifact from the environment^28^.

To test the selectivity of our sensor, the peptide aptamer-modified SWCNT FETs were subjected to 1 ng/mL BSA for 10 min, and 0.1 and 1 ng/mL CatK for 10 min, separately, rinsed thoroughly with 0.0005× PBS pH 4 and then dried with nitrogen gas prior to collection of their transfer curves.

The non-specific binding of CatE molecules onto SWCNT was examined via an experiment in which the PBASE linker was immobilized onto a SWCNT FET without the peptide aptamer probe molecule, followed by blocking with 100 mM ethanolamine for 30 min. The PBASE linker-modified SWCNT FETs were then subjected to 1 ng/mL CatE for 10 min, rinsed thoroughly with 0.0005× PBS pH 4 and then dried with nitrogen gas prior to collection of their transfer curves.

### AFM preparation of CatE captured on the peptide aptamer-modified SWCNT FET

The 10 ng/mL CatE in 1× PBS pH 10 was dropped onto the peptide aptamer-modified SWCNT FET and then incubated at 25 °C for 10 min, rinsed completely with 0.0005× PBS pH 4 and dried with nitrogenous gas. The morphology of bare SWCNT, peptide aptamer-modified SWCNT and CatE-captured peptide aptamer-modified SWCNT FETs were visualized using AFM images.

### Quantitative detection of CatE in 10-fold-diluted human serum

The peptide aptamer-modified SWCNT FETs were exposed to various CatE concentrations (from 10 ng/mL to 100 ng/mL), which were prepared in 10-fold diluted human serum and then incubated for 20 min at 25 °C, rinsed thoroughly with milli-Q water and 0.0005× PBS pH 4 and then dried with nitrogen gas. The transfer characteristics of the sensors were measured in 0.0005× PBS pH 4 for each CatE concentration using the top liquid-gated scheme.

## Electronic supplementary material

Supplementary Information
