# Supplementary material for: Peptide aptamer-modified single-walled carbon nanotube-based transistors for high-performance biosensors
Source: Sci Rep. 2017 Dec 20;7:17881. doi: 10.1038/s41598-017-18169-1 (PMC5738443; doi:10.1038/s41598-017-18169-1)

**Supplementary Information**

**Peptide aptamer-modified single-walled carbon nanotube-based transistors for high-performance biosensors**

**Nguyen Thanh Tung^1^, Phan Trong Tue^1^, Truong Thi Ngoc Lien^2^, Yasuhide Ohno^3^, Kenzo Maehashi^4^, Kazuhiko Matsumoto^5^, Koichi Nishigaki^1^, Manish Biyani^1^ & Yuzuru Takamura^1^**

^1^School of Materials Science, Japan Advanced Institute of Science and Technology, 1-1 Asahidai, Nomi city, Ishikawa 923 - 1292, Japan

^2^School of Engineering Physics, Hanoi University of Science and Technology, 1 Dai Co Viet Road, Hanoi, Vietnam

^3^Graduate School of Technology, Industrial and Social Sciences, Tokushima University, 2-4 Shinkuracho, Tokushima 770 - 8501, Japan

^4^Institute of Engineering, Tokyo University of Agriculture and Technology, 2-24-16 Nakacho, Koganei, Tokyo 184 - 8588, Japan

^5^The Institute of Scientific and Industrial Research, Osaka University, 8-1 Mihogaoka, Ibaraki, Osaka 567 - 0047, Japan

^6^Department of Functional Materials Science, Saitama University, 255 Shimo-okubo Sakura-ku, Saitama city, Saitama 338-8570, Japan

^7^Center for Single Nanoscale Innovative Devices, Japan Advanced Institute of Science and Technology, 1-1 Asahidai, Nomi, Ishikawa 923-1292, Japan

Corresponding author(s):

Manish Biyani, PhD

E-mail: [biyani@jaist.ac.jp](mailto:biyani@jaist.ac.jp); Tel. +81-761-51-1591, Fax: +81-761-51-1665

Yuzuru Takamura, PhD

E-mail: [takamura@jaist.ac.jp](mailto:takamura@jaist.ac.jp); Tel. +81-761-51-1661, Fax: +81-761-51-1665

**Supplementary Figure S1:** Output characteristics of fabricated SWCNT FET using (a) back-gated and (b) top-liquid-gated schemes. In both measurements, the source-drain voltage was scanned from -0.1 V to 0.1 V while the gate voltages were varied from -5 V to 3 V and from -0.6 V to 0 V for the back-gated and liquid-gated devices, respectively. At V_DS_ of 0.1 V, the drain currents decreased with increasing gate voltage, which represented the characteristic of typical p-type SWCNT FETs for the both back-gated and liquid-gated devices.


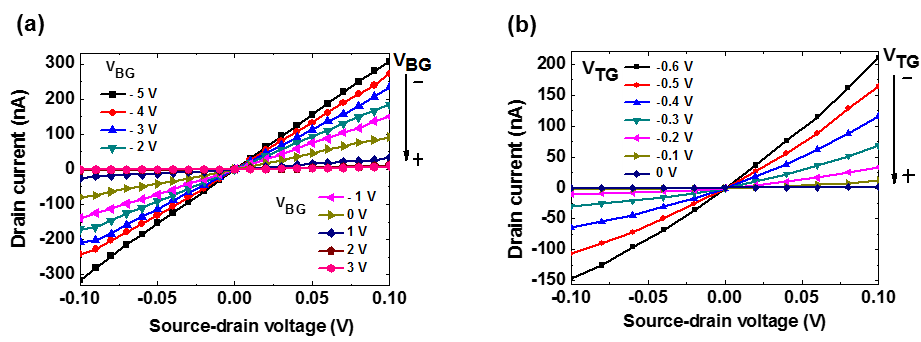


**Supplementary Figure S2:** Operation stability of SWCNT FET in air versus in liquid. The transfer characteristics of fabricated SWCNT FET with 10 measurements, using (a) back-gated scheme and (b) top liquid-gated scheme. The variation of transfer curves (∆V) at drain current of 80 nA are 345 mV and 29 mV, corresponding to back-gated and top liquid-gated scheme, respectively. The result indicated that the fabricated SWCNT FET manifested stable operation in liquid-gated scheme rather than in ambient air scheme.


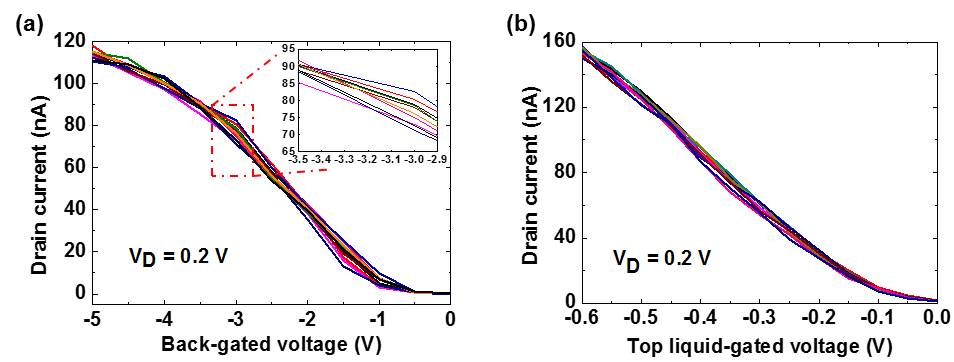


**Supplementary Figure S3:** The investigation of (a) PBASE linker concentration; and (b) peptide aptamer concentration.


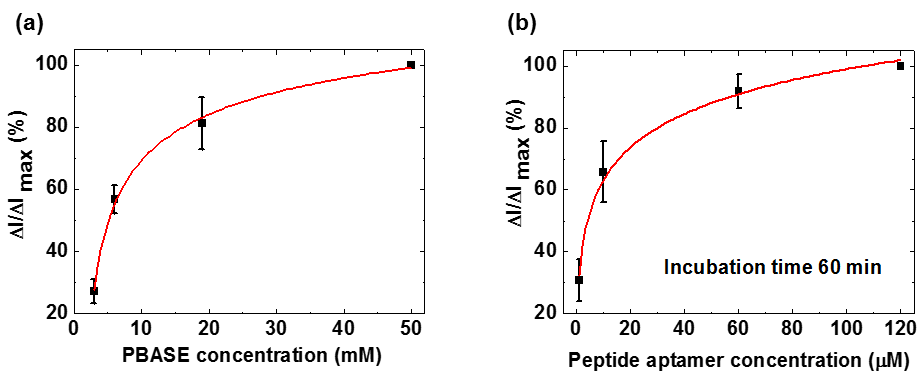


**Supplementary Figure S4:** The AFM microphotographs of the device before and after capture of CatE. (a) Bare SWCNT, (b) peptide aptamer-immobilized SWCNT and (c) CatE captured on the peptide aptamer that was immobilized on the SWCNT.

**
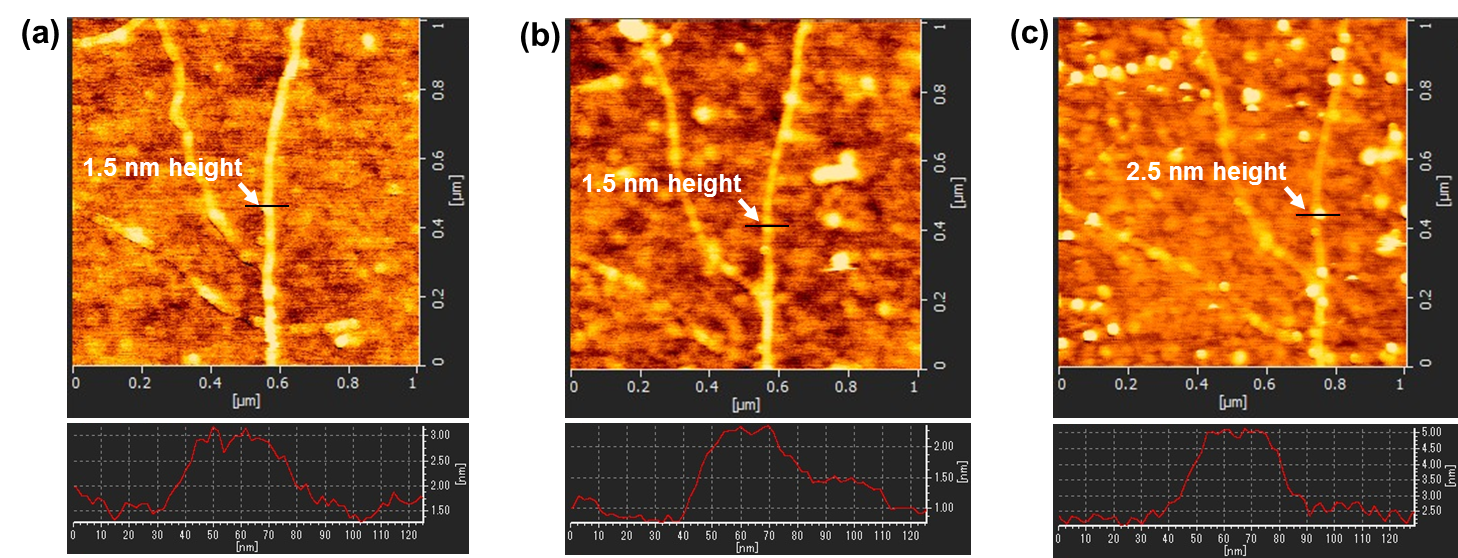
**

**Supplementary Figure S5:** SWCNT synthesis process.


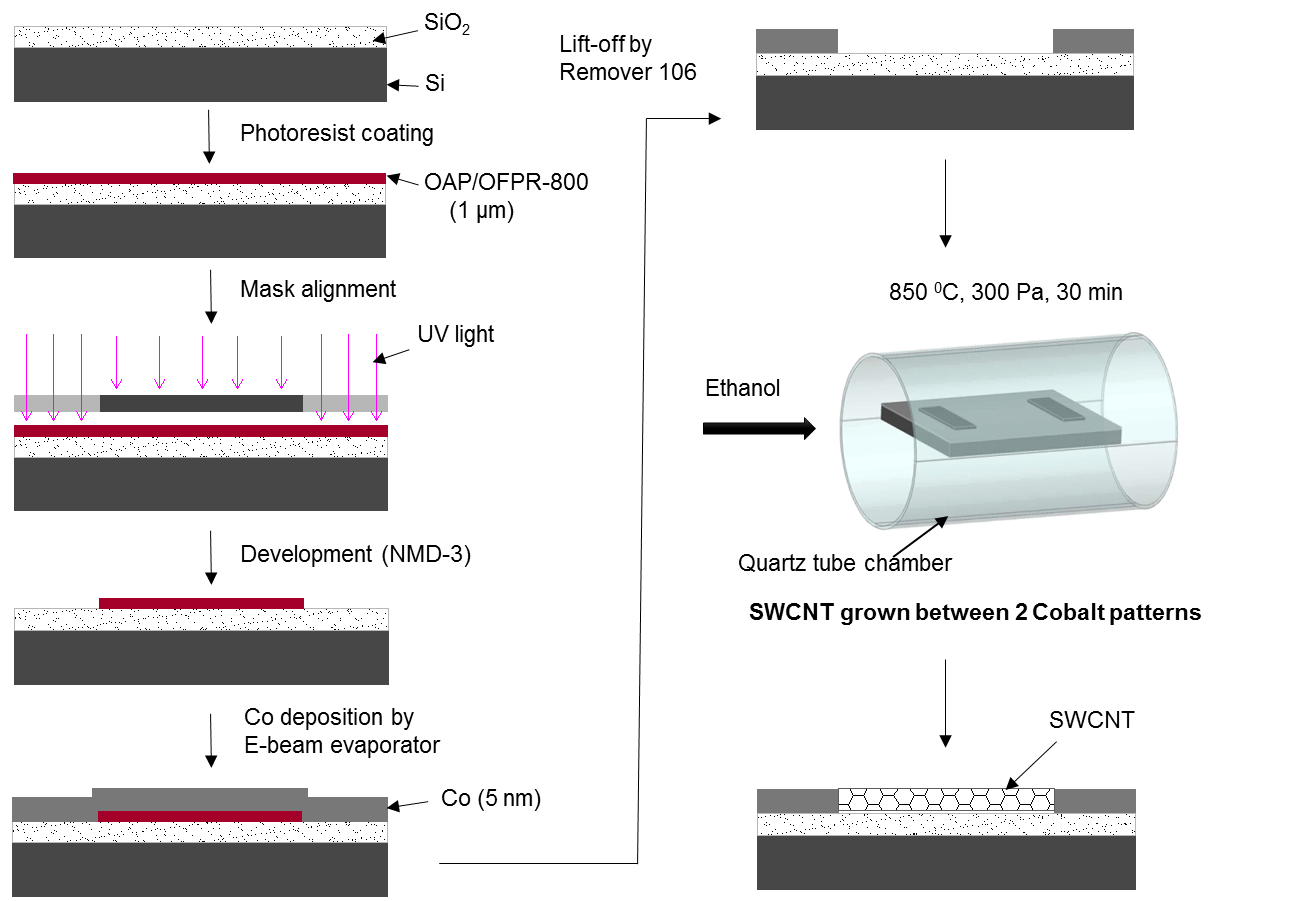


**Supplementary Figure S6:** FET fabrication process.


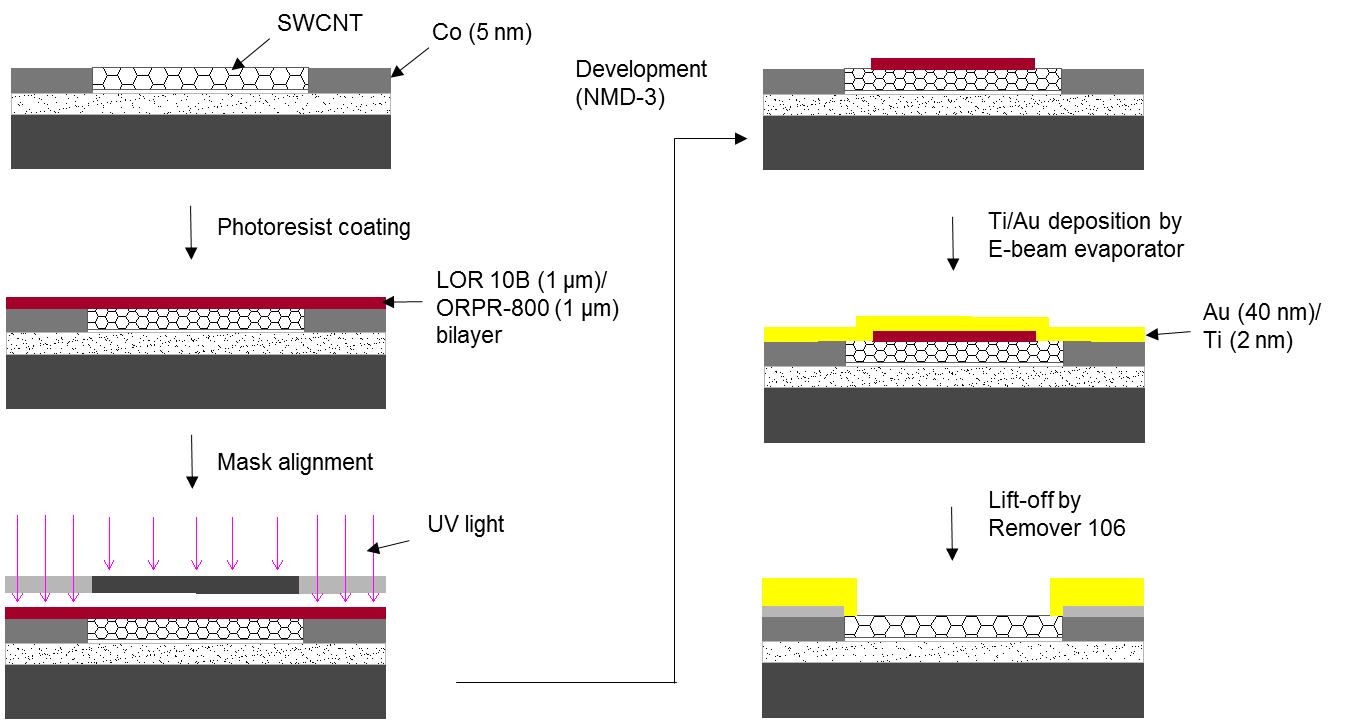

Supplement: Supplementary file 1 — Supplementary Information [file 41598_2017_18169_MOESM1_ESM.doc]
